# Effective Utilization of Waste Glass as Cementitious Powder and Construction Sand in Mortar

**DOI:** 10.3390/ma13030707

**Published:** 2020-02-05

**Authors:** Yanru Wang, Yubin Cao, Peng Zhang, Yuwei Ma

**Affiliations:** 1 Centre for Durability & Sustainability Studies of Shandong Province, Qingdao University of Technology, Qingdao 266033, China; yanru.wang@usq.edu.au (Y.W.); yubin.cao@usq.edu.au (Y.C.); 2 Centre for Future Materials, University of Southern Queensland, Toowoomba QLD 4350, Australia; 3 Guangzhou University-Tamkang University Joint Research Center for Engineering Structure Disaster Prevention and Control, Guangzhou University, Guangzhou 510006, China; yuwei.ma@usq.edu.au

**Keywords:** waste glass powder, waste glass sand, alkali–silica reaction, active SiO_2_

## Abstract

The purpose of this study is to investigate the availability of waste glass as alternative materials in sustainable constructions. Collected waste glass was ground into waste glass powder (WGP) with similar particle size distribution as Portland cement (PC) and waste glass sand (WGS) with similar grade as sand. The compressive strength was investigated through the Taguchi test to evaluate the effect of different parameters on WGP-blended mortar, which include WG-replacement rate (G/B, 0, 10%, 20%, 30%), water/binder ratio (w/b, 0.35. 0.40, 0.50, 0.60), cementitious material dosage (*Cpaste*, 420, 450, 480, 500 kg/m^3^), and color of powder (green (G) and colorless (C)). The alkali–silica reaction (ASR) expansion risk of WGS-blended mortar was assessed. The experimental results indicated that WGP after 0.5 h grinding could be used as substituted cement in mortar and help to release potential ASR expansion. The replacement rate played a dominant role on strength at both the early or long-term age. The water/binder ratio of 0.35 was beneficial to the compressive strength at three days and 0.50 was better for strength at 60 and 90 days. An optimal value of cementitious material dosage (450 Kg/m^3^) exited in view of its strength, while the effect of the color of WG was minor. WGS could be graded as standard construction sand and no ASR expansion risk was found even for 100% replacement of regular sand in mortar. Through the comprehensive reuse of waste glass, this study could provide basic knowledge and a concept for the sustainable development of building materials.

## 1. Introduction

Portland cement has been the most popular construction material for a century [1,2]. However, the environmental impact during cement production, for example, emissions of greenhouse gas, has also been frequently concerned [3]. Sustainable development in the cement industry encouraged the development of supplementary cementitious materials (SCMs) that not only improve the workability [4], durability [5,6], and strength of concrete, but also reduce the consumption of Portland cement per unit volume of concrete [7,8]. Actually, a wide range of by-products, such as fly ash, blast furnace slag, or rice husk ash are commonly used as SCMs [7]. The main chemical compositions of SCMs are aluminosilicate (SiO_2_-Al_2_O_3_) or calcium aluminosilicate (CaO-SiO_2_-Al_2_O_3_) [9].

Except for the aforementioned materials, the growing demand of SCMs is pushing industry and academia to explore more alternatives from a practical point of view [10], for example, waste glass (WG). Owing to the stable chemical properties of WG, it takes thousands of years to degrade completely after landfill. It has been proven that the best way to dispose of WG is to recycle it [11]. The main chemical composition of WG is silicon dioxide (SiO_2_) and various metal oxides, that is, Ca, Al, and Fe, among others [12]. In theory, it can be fully recycled within its usual manufacturing process [13]. However, this is not an easy task because of the complicated process of cleaning, separating, and sorting [14]. A major effort has been made in academia for decades to develop WG as a construction material [15,16,17,18,19].

WG was firstly proposed as a coarse aggregate in construction concrete. By optimizing the mixture proportion, the negative effect of WG on mechanical strength can be predicted and accepted. However, non-negligible alkali–silica reaction (ASR) expansion was the main concern for re-utilization of WG. This is because active silicon oxide (SiO_2_) in WG could react with calcium hydroxide solution (Ca(OH)_2_) to generate an expansive reaction product [20]. Johnston [21] found strength regression and excessive expansion due to ASR in concrete containing glass crushed to 19 mm. However, they also proposed that the reducing amount of cement appeared to alleviate the ASR and improve long-term performance. At Columbia University, broad studies were also carried out to solve ASR problem through the reuse of waste glass as an aggregate in paving, blocks, tiles, and precast panels [22]. Ducman, et al. [23] reheated the mixture of WG together with an expansive agent to produce lightweight WG aggregate with a concluded porous structure to reduce the ASR. Park, et al. [24] identified the decrease in compressive strength of concrete containing a higher replacement rate of WG as a fine aggregate. However, he also identified the positive effect of WG on slump and air content. Mardani-Aghabaglou, et al. [25] displayed the improvement of the freeze–thaw resistance of mortar containing WG aggregates owing to the generation of more pores.

Considerable waste glass powders (WGPs) were generated during the grinding process. Recently, support has been growing for the utilization of WGP as SCMs to replace part of ordinary Portland cement (OPC). Owing to the complex sorting process and similar composition character, mixed colorful WGP was usually used as SCMs in previous research [18,26]. Shi, et al. [27] studied the pozzolanic activity of four glass powders: crushed WGP, glass dust from crushing process, and two dusts from further grinding. They found that finer glass powder possessed higher pozzolanic reactivity. When used as SCMs in blended cement mortar, it could reduce the potential of ASR expansion. Shayan and Xu [28] tried to use WGP to replace OPC in a field trial on concrete slabs (1.5 m × 2.5 m × 0.25 m), and found that WGP could be incorporated into 40 MPa concrete with a 20–30% replacement rate and enhance the chloride resistance. The works of [29] and [30] further confirmed the role of WGP in improving the durability of mortar and concrete. Islam, Rahman, and Kazi [26] found that 1% super plasticizing admixture in the mixture could help to improve the compressive strength of mortars, displaying the non-ignorable effect of water/binder in properties of samples with GP. Researchers also attempted to utilize WGP as raw materials for alkali-activated mortars or concrete [31,32,33,34]. However, the activators used in alkali-activated materials still have environmental and economic impact owing to high emissions and energy consumption associated with the industrial production of sodium silicate [35].

To the best of our knowledge, previous works have focused on the effect of the replacement rate of WG to cement or aggregates. From the previous results, we can observe the non-negligible effect of the cement dosage and the water/binder ratio on properties of WGP blended samples. In this study, three waste glasses with different colors were collected and tested. Firstly, the pozzolanic activity of WGPs and its effect on ASR expansion was evaluated. Then, a Taguchi test was designed to assess the effect of water/binder (w/b, 0.35. 0.40, 0.50, 0.60), cementitious material dosage (*Cpaste*, 420, 450, 480, 500), replacement rate (glass/cement (G/B), 0%, 10%, 20%, 30%), and color of glass powder (green and colorless). Finally, glass culets were ground and graded into waste glass sand (WGS) according to American Society for Testing and Materials (ASTM) C1260 [36], and then the potential ASR expansion was tested.

## 2. Materials and Experimental Methods

### 2.1. Raw Materials

#### 2.1.1. Cement

Ordinary Portland cement (OPC) Type 42.5 (Shanshui, Qingdao, China) was used for the activity rate index test and Taguchi mixture design. Low alkali cement Type 52.5 (Na_2_O + 0.658K_2_O ≤ 0.6%, Shanshui, Qingdao, China) was used for the volume stability test to reduce ASR expansion caused by OPC to the greatest extent possible. The chemical composition as well as physical and mechanical properties of these two PCs are given in Table 1. International Organization for Standardization (ISO) standard sand (GSB08-1337, Aisiou, China) was used as normal standard sand.

#### 2.1.2. Waste Glass Powder (WGP)

Colorless glass from a white wine bottle, green glass from a beer bottle, and brown glass from a chemical reagent bottle were sourced from post-consumer bottles in Qingdao. After being rinsed with tap water, the glass cullet was dried at 100 °C in an oven overnight. The glass cullet was ground with a Planetary ball mill for 0.5 h, 1 h, 1.5 h, 2 h, and 2.5 h, respectively, and then the waste glass powder was collected after passing through a 75 µm sieve. The composition of green glass powder (GP), colorless glass powder (CP), and brown glass powder (BP) after 2.5 h milling is shown in Table 2. Different color glass powders had minor difference in chemical composition, while the SiO_2_ amount was over 70% in BP. Islam, Rahman, and Kazi [26] also found a minor difference in the X-ray diffraction (XRD) result among different color glass powders. Glass powder showed a lower SiO_2_ amount compared with silicon fume, where the SiO_2_ amount of silicon fume as a supplementary cementitious material was around 80% [37]. However, silicon fume had a negative effect on alkali–silica reactivity and during shrinkage [38].

#### 2.1.3. Waste Glass Sand (WGS)

WGS was prepared according to the Chinese standard for building sand GB/T 14684-2011 [39,40]. The glass cullet was sieved and classified by different sizes, for example, 4.75–2.36 mm, 2.36–1.18 mm, 1.18–0.6 mm, 0.6–0.3 mm, and 0.3–0.15 mm. Then, according to cumulative percentage, the WGS was prepared as shown in Table 3.

### 2.2. Research Framework

**Figure 1:**
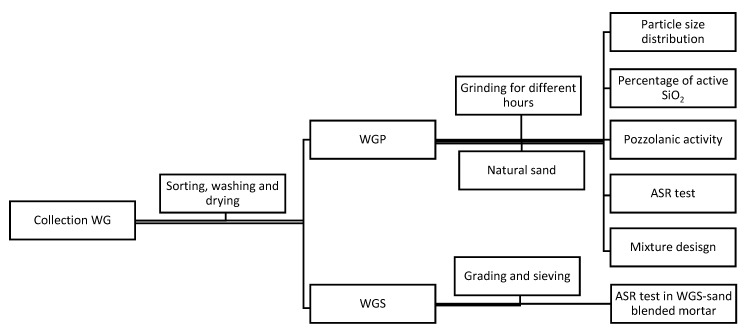
The research framework. WGS, waste glass sand; WGP, waste glass powder; ASR, alkali–silica reaction.

To effectively utilize WG as construction materials, WG was separated into two parts: fine powders (WGP) as supplementary cement and coarse particles (WGS) as construction sand. Before evaluating the effect of different parameters on the optimal mixture design of WGP blended mortar, chemical properties (i.e., percentage of active SiO_2_ and pozzolanic activity) and the physical property (i.e., particle size distribution) were recorded. The ASR test of WGP blended mortar could help to verify the suitability of the chosen grinding time. The last part aimed to check the potential ASR of WGS as supplemental fine aggregate in mortar. ISO standard sand was used as the control group. Figure 1 shows the overview of the research framework.

### 2.3. Experimental Methods

#### 2.3.1. Alkali–Silica Reaction (ASR) Test

The volume stability of WGS and WGP mortars was measured in this contribution to reflect the ASR risk (shown in Table 4). The volume stability test was conducted at the standard test room, where the temperature was kept at 20~27.5 °C and the relative humidity was less than 80%. The test procedure followed Standard Test Method for Potential Alkali Reactivity of Aggregates (Mortar-Bar Method) (ASTM C1260-07). The expansion rate of samples can be calculated using Equation (1):

(1) $$\sum_{t}=\frac{L_{t}-L_{0}}{L_{0}-2\Delta }$$

where: ∑*_t_* is the expansion rate of test samples at *t* age; *L_t_* is the length of the test samples at *t* age; *L*_o_ is the original length of the test samples before the test; and ∆ is the length of the expansion probe. The judgment criteria for the ASR risk were based on GB/T14684-2001 and ASTM C1260-07, as follows:

1. When ∑_16_ ≤ 0.10%, in most cases, it can be judged that there is no potential ASR hazard;
2. When ∑_16_ ≥ 0.20%, it can be determined that there is a potential ASR hazard;
3. When ∑_16_ is between 0.10% and 0.20%, the potential ASR hazard cannot be finally determined, while supplementary information should be supplied.

#### 2.3.2. Pozzolanic Activity Test

##### SiO_2_ Activity Rate (Ka)

The pozzolanic activity (*Ka*) was measured according to the content of active SiO_2_ of WGPs, as the low content contribution of Al_2_O_3_ and Fe_2_O [27,41]. An improved method was adopted in this contribution. Active SiO_2_ could react with Ca(OH)_2_ (2), and the reaction product (i.e., Ca_3_Si_2_(OH)_3_) dissolved in hydrochloric acid (HCl) solution (3). The reaction product (SiCl_4_) could react with sodium hydroxide (NaOH) solution to achieve flocculated white precipitate, that is, H_2_SiO_3_ (4). After calcined above precipitate at 950 °C for 2 h, the weight of active SiO_2_ could be calculated (5).

Glass powder (Active SiO_2_) + Ca(OH)_2_ (saturated) − Ca_3_Si_2_(OH)_3_ + Residue (2)

Ca_3_Si_2_(OH)_3_ + HCl (~3 mol/l) − CaCl_2_ + SiCl_4_ + H_2_O + Residue (3)

SiCl_4_ + NaOH − NaCl + H_2_SiO_3_ (flocculated white precipitate) + H_2_O. (4)

m(SiO_2_) = M(SiO_2_)/M(H_2_SiO_3)_ * m(H_2_SiO_3_) = 60.083/78.098 * m(H_2_SiO_3_) (5)

The detailed steps are listed below:

Prepare WGP. WGP was dried at 105 °C oven until weight remained constant. Prepared 0.500 g WGP as one sample.

Prepare saturated Ca(OH)_2_ and NaOH solution. Ca(OH)_2_ powder was dissolved in distilled water until no more dissolution occurred. Saturated NaOH solution was prepared as it is. It was allowed to stand still until the precipitate sunk to the bottom of the volumetric flask. The supernatant was the saturated Ca(OH)_2_ and NaOH solution.

Mixed WGP and saturated Ca(OH)_2_ solution (Solution 1). To accelerate the full reaction between active SiO_2_ and Ca(OH)_2_, alcohol lamp boiling and condensing reflux were applied in this contribution.

Concentrated hydrochloric acid titration. Then, 8 mL HCl solution (12 mol/L) was added into the above cooling Solution 1 to get Solution 2. The, it was boiled again for 5 min, cooled down, and allowed to stand still. Solution 3 was obtained by filtering Solution 2.

Saturated NaOH solution titration. Saturated NaOH solution was added into the above Solution 3 until no more flocculated white precipitate occurred. It was filtered again and the filter paper and sediment were kept.

High temperature furnace ashing. The filter paper and sediment were placed in high-alumina crucible, and then the crucible was placed in a high-temperature furnace. Then it was calcined at a set temperature of 950 °C for 2 h, including heating for 1 h while warming up. The residue was pure H_2_SiO_3_.

##### Activity Rate Index (Kc)

The compressive strength in the name of the activity rate index (*Kc*) of WGP was measured according to ASTM C 618. Mortar samples (40 mm × 40 mm × 160 mm) were prepared using 70% OPC and 30% WGP. The ratio of water/binder and binder/sand was 0.5 and 0.33, respectively, with a contrast sample that consisted of 100% OPC. *Kc* could be calculated using Equation (6), where *f_cn_* was the compressive strength of mortar after *n* days curing and *f_c_*_0_ was the compressive strength of contrast sample at same curing age:

(6) KC=fcufc0×100%

#### 2.3.3. Taguchi Design for WGP Blended Mortars

The Taguchi experiment design is a fractional factorial design method that use orthogonal arrays to reduce test numbers on the basis of investigation of a large number of variables [42].

In this contribution, four factors were investigated, that is, the water/binder ratio (w/b, 0.35. 0.40, 0.50, 0.60), cementitious material dosage (*C_paste_*, 420, 450, 480, 500 kg/m^3^), replacement rate (glass/cement ratio (G/B), 0%, 10%, 20%, 30%), and color of glass powder (green, white, green, green [43]), as shown in Table 5. According to the Taguchi experiment design, 16 trials were prepared depending on the L16 (4^4^) array. The component parameters for each trial mixture are given in Table 6. Compressive strength (40 mm × 40 mm × 160 mm samples) was measured at the curing age of 3 d, 7 d, 28 d, 60 d, and 90 d (20 ± 3 °C, relative humidity (RH) ≥ 95%), respectively.

The *K* value is the average value of each factor with same level; the *R* value is the range of *K* value at each factor. The key factors and the effect of each factor could further analyzed based on the *K* value and *R* value [44]. The *K* value and *R* value could help to find the dominant factor that affect the strength performance of WGP blended mortar. This contributed to figuring out the optimum combination of factors and levels [44]. However, the result is not very accurate, and more details and the accurate effect of each factor should be explored in further study.

#### 2.3.4. Thermogravimetric Analysis-Derivative Thermogravimetry (TGA-DTG) Test

TGA-DTG test is an analytical method recording the relationship (i.e., thermogravimetric curve) between mass and temperature on a thermal balance. The following is a detailed operating procedure: turn on the TA Instruments TGA Q 650; adjust the pressure of the nitrogen bottle to 0.1–0.2 MPa; balance the empty crucible, and add around 10–15 mg sample powder into the balanced crucible; set the temperature from room temperature to 1400 °C; start the test. After the differential quotient with temperature, a differential quotient thermogravimetric curve (DTG) was obtained. The weight change percentage of calcium hydroxide in samples was determined by the tangent method [45,46]. The decomposition of the relative molar was determined by dividing the lost mass by the relative molecular weight.

## 3. Results and Discussions

### 3.1. Pozzolanic Activity Rate

#### 3.1.1. Percentage of Active SiO_2_ (Ka) in WGP

**Figure 2:**
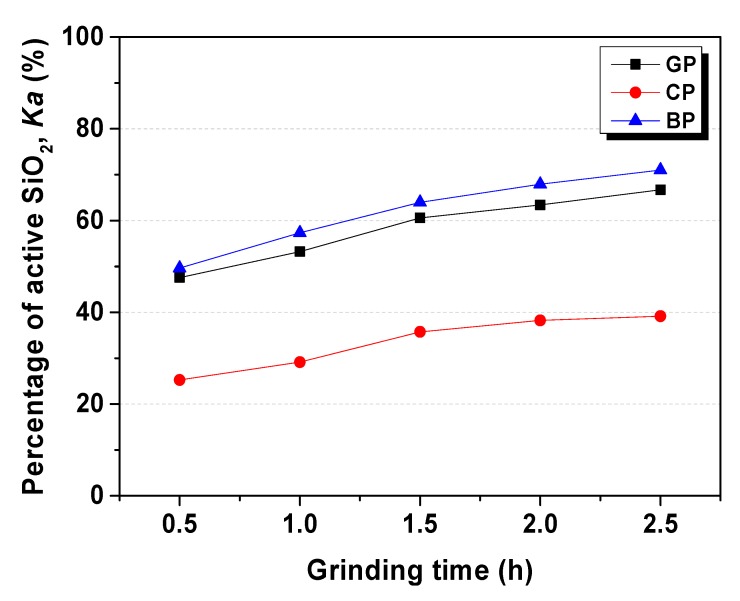
SiO_2_ activity rate of waste glass powder. GP, green glass powder; CP, colorless glass powder; BP, brown glass powder.

The percentage of active SiO_2_ (*Ka*) was measured according to the method mentioned in Section 2.3.2 and recorded in Table 7, Table 8 and Table 9. The active SiO_2_ amount increased continually with the grinding time, as shown in Figure 2. There was a big gap between BP/GP and CP, which *Ka*(BP) closed to *Ka*(GP), although it was much higher than *Ka*(CP). However, the total amount of SiO_2_ in CP was highest from the XRF result (Table 2), which indicted that even colorless glass powder contained more SiO_2_, and just a few parts of SiO_2_ presented chemical activity, in the presence of alkali solution.

#### 3.1.2. Particle Size Distribution of Three WGPs

**Figure 3:**
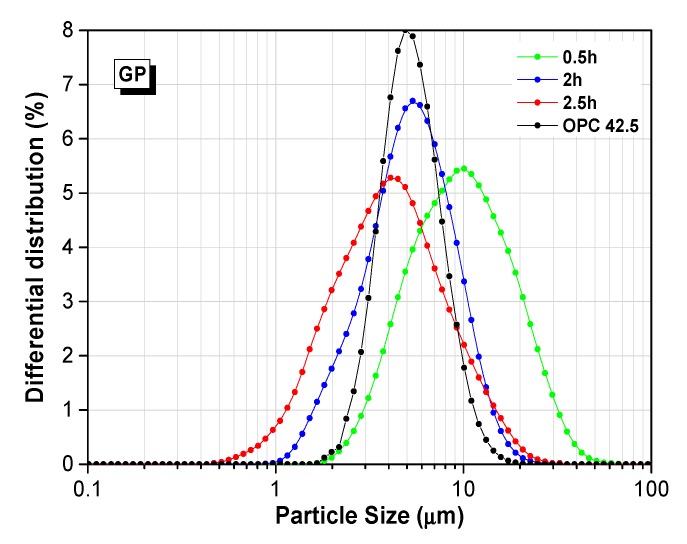

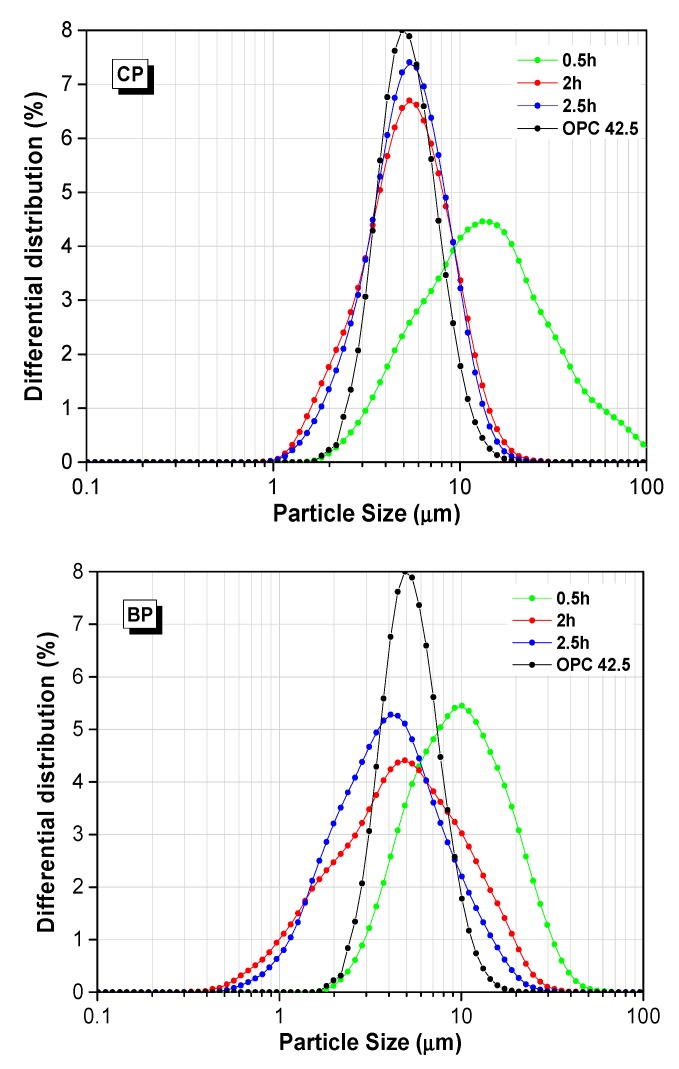
Particle size distribution of GP, CP, and BP after mailing for 0.5 h, 2 h, and 2.5 h. OPC, ordinary Portland cement.

The particle size distribution of WGPs after ball milling for 0.5 h, 2 h, and 2.5 h is given in Figure 3. With the increase of grinding time, the main peak of the distribution curve moved to the left, indicating the increasing percentage of smaller particles in powders. After 2 h grinding, the particle size was less than 20 micron for both GP and CP. Especially, CP possessed a similar particle size distribution after 2 h and 2.5 h grinding. BP presented a slightly rough distribution compared with the other two powders. The particle size distribution of type 42.5 Portland cement was plotted in Figure 3, shown in black. In this contribution, a relatively fine and uniform Portland cement was used because of errors caused from raw materials. Researchers reported the particle size distribution of 20 cements from large cement companies in China [47]. Compared with the previous results of scholars, GP-0.5 h and BP-0.5 h had a similar particle size distribution as most Chinese Portland cement [47]. Therefore, the particle size of 0.5 h grinding WGP was acceptable as supplementary cementitious materials to substitute PC. Referencing the results of the activity test, although 2 h grinding could increase the active amount by 33% for GP, taking into account the influence of fineness and energy cost, 0.5 h grinding powder was used for the following pozzolanic activity test, ASR test, and Taguchi test.

#### 3.1.3. Pozzolanic Activity Rate of WGP (Kc)

**Figure 4:**
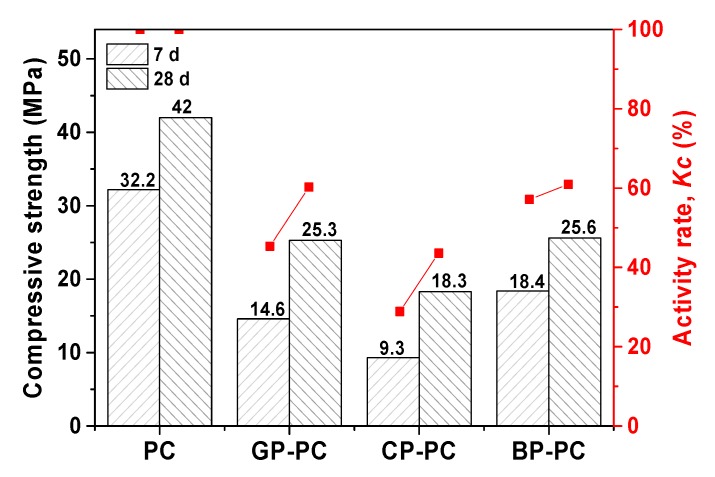
The activity rate (*Kc*) of glass powder as supplementary cementitious materials as repented by compressive strength change.

The pozzolanic activity rate (*Kc*) of WGP was given based on the change of compressive strength. The samples were prepared with 70% PC and 30% glass powder. From Figure 4, it was worth noting that the decrease of compressive strength happened after glass powder substituted cement. Similar results also were demonstrated in previous studies [12,24,26]. This was because that the weak bond between non-active silicon with the cement matrix would affect the strength [48]. The activity rate for three glass powders increased after 28 days’ curing. The *Kc* of the three glass powders under the same curing age is brown > green > colorless. It was assumed that the active SiO_2_ was one of the reasons. The influence was significant at an earlier age, rather than after 28 days’ curing.

### 3.2. ASR Test of WGP-Cement Mortar

**Figure 5:**
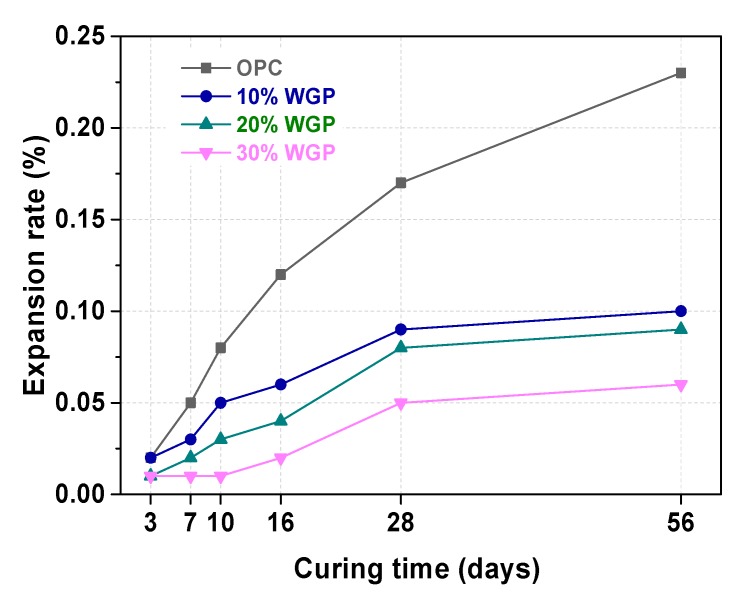
Expansion rate of ASR of mortars with WGP substituting 0%, 10%, 20%, and 30% of Portland cement.

As shown in Figure 5, the expansion rate of mortars extended with the increase in curing time. According to the mortar bar method, the OPC mortars (i.e., 0% WGP) showed a 0.12% expansion rate (0.10% ≤ ∑*_16_* ≤ 0.20%) at 16 days of age, meaning it is hard to determine the risk of ASR. The samples containing 10% WGP, 20% WGP, and 30% WGP showed a lower expansion rate than OPC of 0.08%, 0.05%, and 0.02%, respectively. These results suggested that there was no ASR risk of mortars with the partial replacement of WGP. With the increasing substituted ratio of WGP, the relevant expansion rate decreased. This was because WGP could help to reduce the pH inside mortar by consuming calcium hydroxide, which was generated by cement hydration, and thereby reducing the ASR risk [49]. Thomas [50] reviewed the effect of other SCMs, such as silica fume, fly ash, slag, and metakaolin on ASR, and found that most SCMs had a similar positive effect on the ASR test owing to the reduction in the concentration of alkali-hydroxides in pore solution. Thus, WGP as a supplementary cementitious material to replace part of OPC could inhibit the ASR potential risk.

### 3.3. Taguchi Test

#### 3.3.1. Compressive Strength of Mixtures

**Figure 6:**
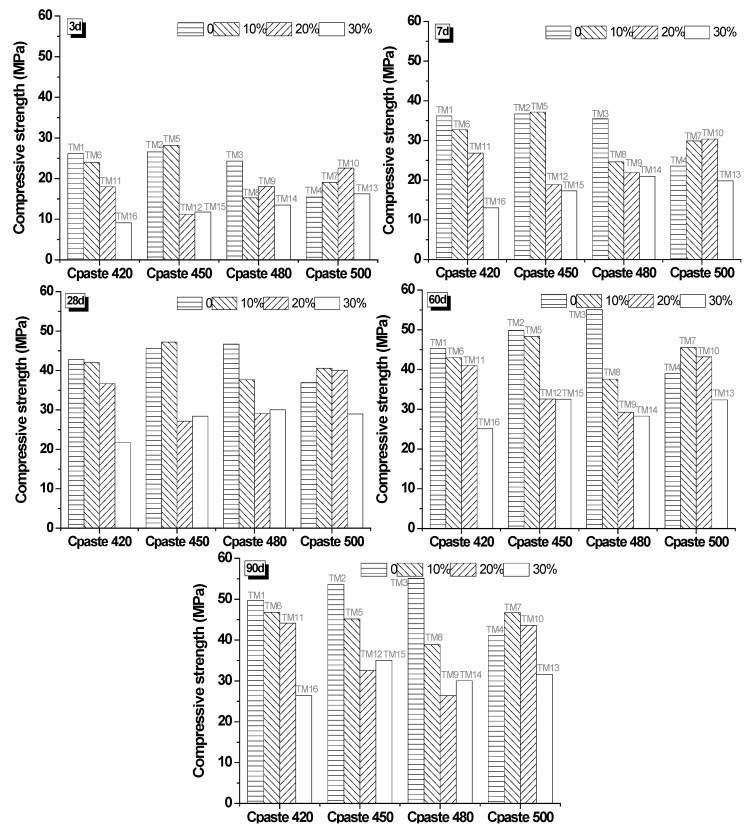
The relationship of substitution rate, curing age, and *C_paste_*.

**Figure 7:**
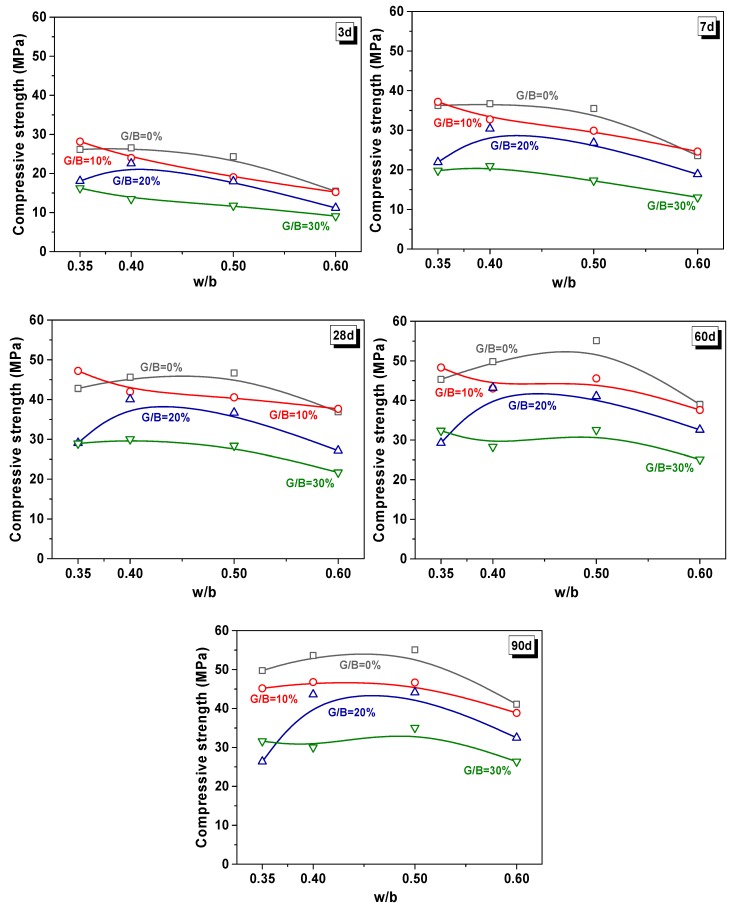
The relationship between the w/b ratio and G/B ratio at different curing ages.

The compressive strength value of mixtures is recorded in both Figure 6 and Figure 7, depending on different relationships. Figure 6 presents the relationship of the WGP substitution rate, curing age, and *Cpaste*. The influence of *Cpaste* was significant on the effect of the substitution rate. When *Cpaste* was 420 kg/m^3^ and 450 kg/m^3^, samples with 10% WGP had comparable strength performance to controls (0% WGP) from the earlier curing age to 90 days. When *Cpaste* was 480 kg/m^3^, there was a big drop from controls to WGP blended samples. After *Cpaste* increased to 500 kg/m^3^, samples with 10% and 20% WGP showed minor higher compressive strength compared with controls. *Cpaste* had a trivial influence on samples with a higher substitution rate (30%). A lower substation of WGP in cement mortar was helpful to improve the compressive strength, which was consistent with previous studies [51,52]. There are two reasons. The first is the pozzolanic reaction between minor active SiO_2_ and Ca(OH)_2_, which was produced by cement hydration [53]. The reaction product of calcium-silicate-hydrate (C-S-H) gel filled the pores and then improved the compressive strength. On the other hand, the superfine particles in the glass powder had the same filler effect on pores [54].

Figure 7 shows the relationship between the w/b ratio and G/B ratio at different curing ages. The compressive strength of samples increased with the extension of the curing age, but tended to slow down after 28 days. The reason is the continuous hydration of cement during the hydration process, which reached the relative highest degree after 28 d of curing [55,56]. It was important to figure out that the coupling effect of the w/b ratio and G/B ratio on compressive strength remained consistent with the increased curing age. The controls and 20% WGP blended samples increased first from w/b = 0.35 to w/b = 0.4, and decreased from w/b = 0.4 to w/b = 0.6. For samples with 10% and 30% WGP, the compressive strength showed a fluctuant drop with the increase of w/b. The discrepancy between samples with 0% to 30% substituted WGP was small at high w/b (i.e., 0.6).

#### 3.3.2. K Value and R Value Analysis

**Figure 8:**
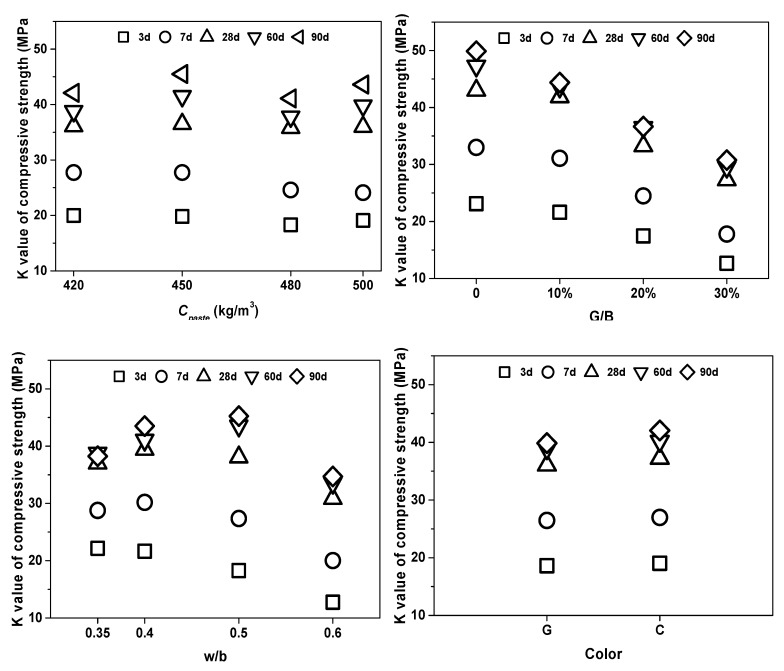
The influence of curing age under different factors.

The effect of the curing age under different factors was plotted through the *K* value (Figure 8). The *K* value of samples at 28 d, 60 d, and 90 d curing age closed to each other, meaning the effect of any factor was significant at the earlier age and trended to mitigation at the later ages. This might be because of the hydration process in the cementitious matrix, which was fast at the earlier age and slowed down with the curing process [55].

**Figure 9:**
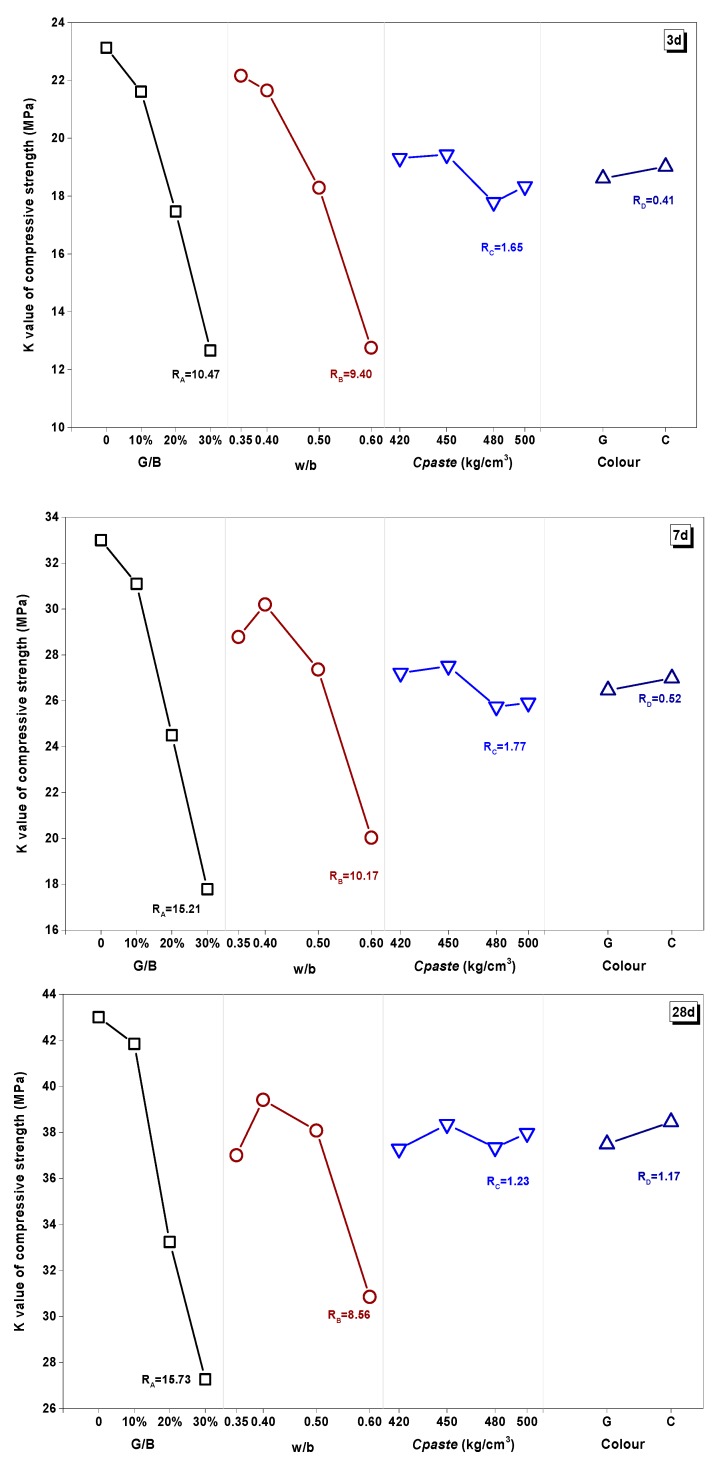

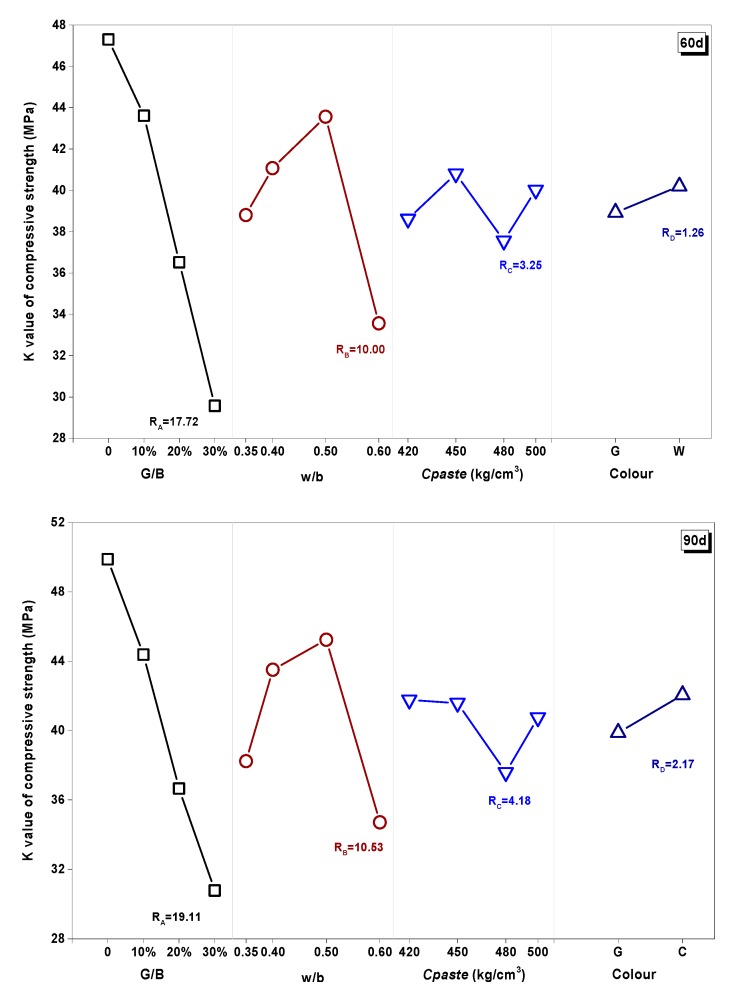
Factorial diagrams of the main parameters affecting the compressive strength of mixtures at 3 d, 28 d, and 90 d.

Figure 9 shows the factorial diagrams of the main parameters affecting the compressive strength of mixtures. The factors and levels had different influences on the strength performance at different curing ages, while *R_A_* ≥ *R_B_* » *R_C_* ≥ *R_D_* at the earlier age. With the prolonging of curing time, the influence proportion (*R* value) of the substituted rate (G/B) gradually deepened from 10.47 to 19.11, while the *R* value of the water/binder (w/b) ratio kept fluctuating around 10. However, the optimal w/b value changed (w/b = 0.35) from low at 3 d to high (w/b = 0.50) at 60 d and 90 d. This result suggested that a lower water/binder ratio had better performance for earlier properties, while a higher and critical value had better performance in the long-term.

The effect of *Cpaste* had a small change with different curing ages. At earlier ages, smaller cementitious dosage displayed better performance. At 28 days, different *Cpaste* had a similar effect, while this effect became significant at 90 days. It was interesting to find that, when *Cpaste* was 480 kg/m^3^, the compressive strength decreased obviously at the long-term curing age. From the above experiments, colorless glass powder with a slightly better strength performance might have contributed to the lower test number. However, the *R* value of color was so small that the influence of color in strength properties could be ignored based on results from this experiment.

#### 3.3.3. Thermogravimetric Analysis (TGA-DTG)

Two mixtures were selected for the TGA test, that is, TM1 (G/B = 0%, w/b = 0.35, *C_paste_* = 420 kg/m^3^) and TM14 (G/B = 30%, w/b = 0.4, *C_paste_* = 480 kg/m^3^, green glass powder). There was a significant loss of weight in different temperature ranges, that is, 120–150 °C for water loss, 440–460 °C for decomposition of Ca(OH)_2_, 700–730 °C for decomposition of CaCO_3_, and 1200–1400 °C for decomposition of sulphate [57], respectively [58,59,60]. The entire process of heating went with the decomposition of calcium-silicate-hydrate (CSH) gel [61,62].

**Figure 10:**
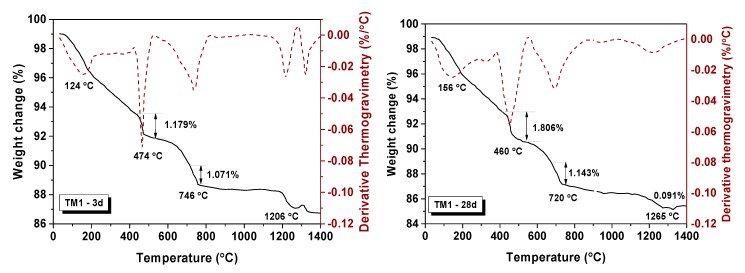
TGA-DTG spectra for TM1-3d and TM1-28d.

**Figure 11:**
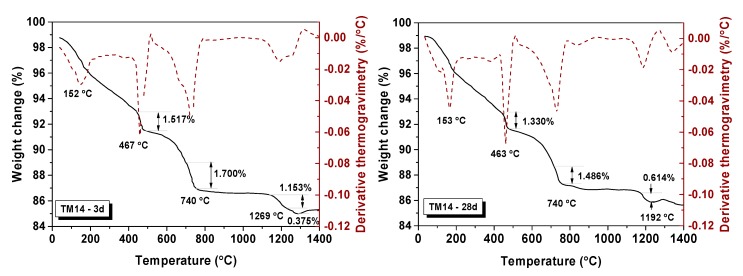
TGA spectra for TM14-3d and TM14-28d.

As shown in Table 10 and Figure 10, about 1.179% of Ca(OH)_2_ was decomposed for samples TM1 cured after 3 days (TM1-3d). This value increased to 1.806% at 28 days. The Ca(OH)_2_ as one of main products of cement hydration continued increasing with the reaction process [63,64]. The amount of CaCO_3_ increased from 1.071% to 1.143% of TM1 owing to the carbonation of Ca(OH)_2_ occurring during the curing. The total molar amount of Ca(OH)_2_ from 3 d to 28 d increased from 0.027 mol to 0.035 mol with the processing of hydration [65]. The Ca(OH)_2_ amount of TM14 (Figure 11) was higher than TM1 after 3 d of curing, as the higher w/b ratio encouraged a fast and quick hydrated reaction [64]. However, the total molar amount of Ca(OH)_2_ decreased from 0.038 mol to 0.033 mol from 3 d to 28 d. This was because active silicon oxide in WGP in mortar could react with Ca(OH)_2_, thereby causing the consumption of Ca(OH)_2_ [66].

### 3.4. ASR Test of WGS-Sand Mortar

**Figure 12:**
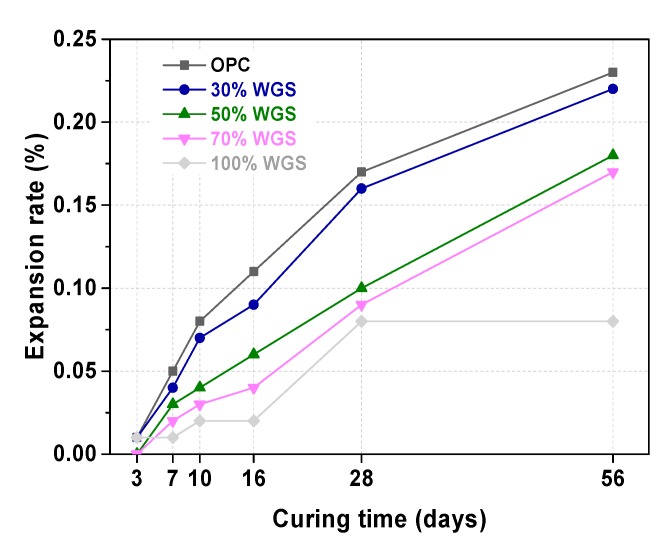
Expansion rate of mortar bars with WGS substituting 0%, 30%, 50%, 70%, and 100% of sand.

**Figure 13:**
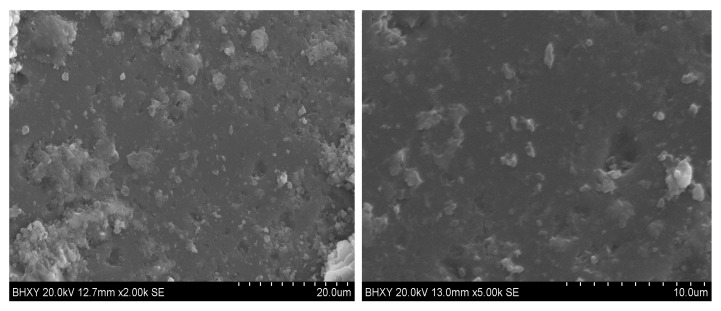
Scanning electron microscopy (SEM) images of green WGS-blended mortar matrix under 2K and 5K magnifications.

As one can see, Figure 12 illustrates the expansion rate of mortars with WGS substituting 0%, 30%, 50%, 70%, and 100% of standard sand. The ∑_16_ of the OPC sample was 0.11%. The WGS substituted samples both displayed minor expansion, less than 0.10%. According to standard specification, the WGS blended samples had no potential ASR risk. Even after 56 days’ curing, the expansion rate of the WGS-blended samples (except 30% WGS) was less than 0.20%. From the collected results, we could infer that the expansion rate of the WGS-blended samples decreased with the amount of the substituted rate (from 10% to 30%). The results obtained from this experiment were contrary to the principle that the increase of the active aggregate content leads to an increase in the expansion rate [49,67]. The reason for the contradiction might be attributed to the microscopic morphology of the samples [68]. Figure 13 shows the microscopic morphology of green WGS blended mortar matrix under different magnifications. From the pictures, we could find a relatively flat surface on the top of WGS. In addition, there are no continued cracks. As a result, there is no place for ASR to occur. However, ASR results were also influenced by replacement ratios [69] and particle size [70].

## 4. Conclusions

The utilization of WGS as construction blended sand in mortar and a composition design of WGP as substituted cementitious materials was presented in this paper. The following conclusions were drawn according to the above results obtained from the specimens and work in this paper:

1. Waste glass could be effectively used as construction materials. The ground glass powder could be used as supplementary cementitious materials. The remaining glass cullet could be used as standard sand.
2. Grinding time played a dominant role in percentage of active SiO_2_ (*Ka)* and particle size distribution of WGP. In terms of green glass powder, 0.5 h of grinding was enough to achieve a suitable particle size distribution to replace PC in mortar.
3. The ASR test results of WGP-blended samples with different substituted rates were encouraging. There was no potential ASR risk presented.
4. The effect of the water/binder (w/b) ratio, cementitious material dosage (*Cpaste*), replacement rate (G/B), and color of glass powder had different impact proportions on the properties of WGP-blended mortars. The effect of *Cpaste* and w/b had a notable difference between the earlier and long-term curing ages. Therefore, in terms of practical application, the optimum design for WGP substituted samples should take account to the influence of curing age.
5. Optimal mixture design could not be addressed from this contribution, owing to the decrease of compressive strength. However, future studies should focus on the optimal mixture design depending on different compressive strength levels.
6. After being graded into standard sand, the WGS could substitute construction sand in cement mortar without an ASR expansion risk.

## Figures and Tables

**Table 1:** Chemical composition (wt. %) as well as physical and mechanical properties of ordinary Portland cement (OPC) Type 42.5 and 52.5 (low alkali cement).

**Material**	**SiO_2_**	**Al_2_O_3_**	**Fe_2_O_3_**	**CaO**	**MgO**	**TiO_2_**	**Na_2_O**	**K_2_O**	**SO_3_**	**LoI**
Type 42.5	20.08	5.09	2.92	61.71	1.57	0.35	0.70	0.36	1.99	5.23
Type 52.5	20.87	4.87	3.59	64.47	2.13		0.11	0.65	2.52	0.77
**Material**	**Setting Time (h)**		**Compressive Strength (MPa)**		**Tensile Strength (MPa)**		**Specific Surface Area (m^2^/kg** **)**			
	**Initial Setting**	**Final Setting**	**3 d**	**28 d**	**3 d**	**28 d**				
Type 42.5	161	217	28.2	41.4	5.4	8.2	350			
Type 52.5	127	128	38.4		7.3		368.9			

**Table 2:** Chemical composition (wt. %) of green glass powder (GP) from X-ray fluorescence (XRF). CP, colorless glass powder; BP, brown glass powder.

Waste Glass Powder	SiO_2_	Na_2_O	K_2_O	CaO	MgO	Al_2_O_3_	Fe_2_O_3_
GP	65.97	11.08	0.35	11.85	1.11	3.33	0.62
CP	68.43	10.97	0.32	11.79	0.91	2.21	0.17
BP	70.69	10.22	1.86	10.79	1.32	3.63	0.52

**Table 3:** Particle size distribution of the waste glass cullet.

Sieve Size (mm)	4.75–2.36	2.36–1.18	1.18–0.6	0.6–0.3	0.3–0.15
Cumulative percentage (%)	10%	25%	25%	25%	15%
Weight (g)	99	247.5	2475	247.5	148.5

**Table 4:** Mixture design for the volume stability test of WGP and WGS (kg/m^3^). WGP, waste glass powder; WGS, waste glass cement.

No.	Portland Cement (PC)	WGP	WGS	Standard Sand	w/b	WGP/PC	WGS/Sand
OPC	440	0		990	0.47	0	
10% WGP	396	44		990	0.47	0.1	
20% WGP	352	88		990	0.47	0.2	
30% WGP	308	132		990	0.47	0.3	
30% WGS	440		297	693	0.47		0.3
50% WGS	440		495	495	0.47		0.5
70% WGS	440		693	297	0.47		0.7
100% WGS	440		990	0	0.47		1.0

**Table 5:** Factors and levels used in the Taguchi experiment design.

No.	Factor	Level 1	Level 2	Level 3	Level 4
A	G/B	0	10%	20%	30%
B	w/b	0.35	0.40	0.50	0.60
C	*C_paste_* (kg/m^3^)	420	450	480	500
D	Color	Green	White	Green	Green

**Table 6:** Taguchi design mixture proportions of samples.

No.	A	B	C	D	Design of Mixture	G/B	w/b	*C_paste_* (kg/m^3^)	Color
TM1	1	1	1	1	A1B1C1D1	0	0.35	420	-
TM2	1	2	2	2	A1B2C2D2	0	0.4	450	-
TM3	1	3	3	3	A1B3C3D3	0	0.5	480	-
TM4	1	4	4	4	A1B4C4D4	0	0.6	500	-
TM5	2	1	2	3	A2B1C2D3	10%	0.35	450	G
TM6	2	2	1	4	A2B2C1D4	10%	0.4	420	G
TM7	2	3	4	1	A2B3C4D1	10%	0.5	500	G
TM8	2	4	3	2	A2B4C3D2	10%	0.6	480	W
TM9	3	1	3	4	A3B1C3D4	20%	0.35	480	G
TM10	3	2	4	3	A3B2C4D3	20%	0.4	500	G
TM11	3	3	1	2	A3B3C1D2	20%	0.5	420	W
TM12	3	4	2	1	A3B4C2D1	20%	0.6	450	G
TM13	4	1	4	2	A4B1C4D2	30%	0.35	500	W
TM14	4	2	3	1	A4B2C3D1	30%	0.4	480	G
TM15	4	3	2	4	A4B3C2D4	30%	0.5	450	G
TM16	4	4	1	3	A4B4C1D3	30%	0.6	420	G

**Table 7:** Percentage of active SiO_2_ of GP.

Milling Time	0.5 h	1 h	1.5 h	2 h	2.5 h
m(H_2_SiO_3_) (g)	0.201	0.225	0.256	0.268	0.282
m(active SiO_2_) (g)	0.156	0.175	0.199	0.209	0.220
m(total SiO_2_) (g)	0.329				
*GP-Ka*	47.6%	53.2%	60.6%	63.4%	66.7%

**Table 8:** Percentage of active SiO_2_ of CP.

Milling Time	0.5 h	1 h	1.5 h	2 h	2.5 h
m(H_2_SiO_3_) (g)	0.111	0.128	0.157	0.168	0.172
m(active SiO_2_) (g)	0.086	0.100	0.122	0.131	0.134
m(total SiO_2_) (g)	0.342				
*WP-Ka*	25.3%	29.1%	35.7%	38.2%	39.2%

**Table 9:** Percentage of active SiO_2_ of BP.

Milling Time	0.5 h	1 h	1.5 h	2 h	2.5 h
m(H_2_SiO_3_) (g)	0.225	0.260	0.290	0.308	0.322
m(active SiO_2_) (g)	0.175	0.202	0.225	0.239	0.250
m(total SiO_2_) (g)	0.353				
*WP-Ka*	49.6%	57.3%	63.9%	67.9%	71.0%

**Table 10:** Decomposition rate (%) of different reaction products.

Samples	Weight Change (%)		Decomposition of Relative Molar (mol)		Total Molar Amount of Ca(OH)_2_ (mol)
	Ca(OH)_2_	CaCO_3_	Ca(OH)_2_	CaCO_3_	
TM1-3d	1.179	1.071	0.016	0.011	0.027
TM1-28d	1.806	1.143	0.024	0.011	0.035
TM14-3d	1.517	1.700	0.021	0.017	0.038
TM14-28d	1.330	1.486	0.018	0.015	0.033

## Abbreviations

ASR	Alkali Silica Reaction
BP	Brown Powder
CP	Colorless Powder
CSH	Calcium Silicate Hydrate
GP	Green Powder
*Ka*	Percentage of Active SiO_2_
*Kc*	Activity Rate of Waste Glass Powder
OPC	Ordinary Portland Cement
SEM	Scanning Electron Microscopy
WG	Waste Glass
WGP	Waste Glass Powder
WGS	Waste Glass Sand
XRF	X-Ray Fluorescence
XRD	X-Ray Diffraction

## References

[1] Aitcin P.C. (2000). Cements of Yesterday and Today: Concrete of Tomorrow. Cem. Concr. Res..

[2] Zhang P., Wittmann F.H., Lura P., Müller H.S., Han S., Zhao T. (2018). Application of Neutron Imaging to Investigate Fundamental Aspects of Durability of Cement-Based Materials: A Review. Cem. Concr. Res..

[3] Biernacki J.J., Bullard J.W., Sant G., Brown K., Glasser F.P., Jones S., Ley T., Livingston R., Nicoleau L., Olek J. (2017). Cements in the 21st Century: Challenges, Perspectives, and Opportunities. J. Am. Ceram. Soc..

[4] Yang X., Liu J., Li H., Ren Q. (2020). Performance and Itz of Pervious Concrete Modified by Vinyl Acetate and Ethylene Copolymer Dispersible Powder. Constr. Build. Mater..

[5] Zhang P., Wittmann F.H., Vogel M., Müller H.S., Zhao T. (2017). Influence of Freeze-Thaw Cycles on Capillary Absorption and Chloride Penetration into Concrete. Cem. Concr. Res..

[6] Bao J., Li S., Zhang P., Ding X., Xue S., Cui Y., Zhao T. (2020). Influence of the Incorporation of Recycled Coarse Aggregate on Water Absorption and Chloride Penetration into Concrete. Constr. Build. Mater..

[7] Lothenbach B., Scrivener K., Hooton R.D. (2011). Supplementary Cementitious Materials. Cem. Concr. Res..

[8] Zhang P., Li D., Qiao Y., Zhang S., Sun C., Zhao T. (2018). Effect of Air Entrainment on the Mechanical Properties, Chloride Migration, and Microstructure of Ordinary Concrete and Fly Ash Concrete. J. Mater. Civ. Eng..

[9] Provis J.L. (2018). Alkali-Activated Materials. Cem. Concr. Res..

[10] Zhu H., Liang G., Zhang Z., Wu Q., Du J. (2019). Partial Replacement of Metakaolin with Thermally Treated Rice Husk Ash in Metakaolin-Based Geopolymer. Constr. Build. Mater..

[11] Kyaw Oo D’Amore G., Caniato M., Travan A., Turco G., Marsich L., Ferluga A., Schmid C. (2017). Innovative Thermal and Acoustic Insulation Foam from Recycled Waste Glass Powder. J. Clean. Prod..

[12] Chandra Paul S., Šavija B., Babafemi A.J. (2018). A Comprehensive Review on Mechanical and Durability Properties of Cement-Based Materials Containing Waste Recycled Glass. J. Clean. Prod..

[13] Butler J.H., Hooper P., Letcher T.M., Vallero D.A. (2011). Chapter 11—Glass Waste. Waste.

[14] Zimmer A., Bragança S.R. (2019). A Review of Waste Glass as a Raw Material for Whitewares. J. Environ. Manag..

[15] Polley C., Cramer S.M., de la Cruz R.V. (1998). Potential for Using Waste Glass in Portland Cement Concrete. J. Mater. Civ. Eng..

[16] Shao Y., Lefort T., Moras S., Rodriguez D. (2000). Studies on Concrete Containing Ground Waste Glass. Cem. Concr. Res..

[17] Topcu I.B., Canbaz M. (2004). Properties of Concrete Containing Waste Glass. Cem. Concr. Res..

[18] Taha B., Nounu G. (2008). Properties of Concrete Contains Mixed Colour Waste Recycled Glass as Sand and Cement Replacement. Constr. Build. Mater..

[19] Jani Y., Hogland W. (2014). Waste Glass in the Production of Cement and Concrete—A Review. J. Environ. Chem. Eng..

[20] Saccani A., Bignozzi M.C. (2010). Asr Expansion Behavior of Recycled Glass Fine Aggregates in Concrete. Cem. Concr. Res..

[21] Johnston C.D. (1974). Waste Glass as Coarse Aggregate for Concrete. J. Test. Eval..

[22] Meyer C.N.E., Andela C. Concrete with Waste Glass as Aggregate. Proceedings of the International Symposium Concrete Technology Unit of ASCE and University of Dundee.

[23] Ducman V., Mladenovič A., Šuput J.S. (2002). Lightweight Aggregate Based on Waste Glass and Its Alkali–Silica Reactivity. Cem. Concr. Res..

[24] Park S.B., Lee B.C., Kim J.H. (2004). Studies on Mechanical Properties of Concrete Containing Waste Glass Aggregate. Cem. Concr. Res..

[25] Mardani-Aghabaglou A., Beglarigale A., Yazıcı H., Ramyar K. (2019). Transport Properties and Freeze-Thaw Resistance of Mortar Mixtures Containing Recycled Concrete and Glass Aggregates. Eur. J. Environ. Civ. Eng..

[26] Islam G., Sadiqul M., Rahman M.H., Kazi N. (2017). Waste Glass Powder as Partial Replacement of Cement for Sustainable Concrete Practice. Int. J. Sustain. Built Environ..

[27] Shi C., Wu Y., Riefler C., Wang H. (2005). Characteristics and Pozzolanic Reactivity of Glass Powders. Cem. Concr. Res..

[28] Shayan A., Xu A. (2006). Performance of Glass Powder as a Pozzolanic Material in Concrete: A Field Trial on Concrete Slabs. Cem. Concr. Res..

[29] Matos A.M., Sousa-Coutinho J. (2012). Durability of Mortar Using Waste Glass Powder as Cement Replacement. Constr. Build. Mater..

[30] Omran A., Tagnit-Hamou A. (2016). Performance of Glass-Powder Concrete in Field Applications. Constr. Build. Mater..

[31] Pascual A.B., Tognonvi M.T., Tagnit-Hamou A. (2014). Waste Glass Powder-Based Alkali-Activated Mortar. Int. J. Res. Eng. Technol..

[32] Redden R., Neithalath N. (2014). Microstructure, Strength, and Moisture Stability of Alkali Activated Glass Powder-Based Binders. Cem. Concr. Compos..

[33] Liu Y., Shi C., Zhang Z., Li N. (2019). An Overview on the Reuse of Waste Glasses in Alkali-Activated Materials. Resour. Conserv. Recycl..

[34] Lu J.-X., Poon C.S. (2018). Use of Waste Glass in Alkali Activated Cement Mortar. Constr. Build. Mater..

[35] Xuan D., Tang P., Poon C.S. (2019). Mswiba-Based Cellular Alkali-Activated Concrete Incorporating Waste Glass Powder. Cem. Concr. Compos..

[36] ASTM C. (2007). 1260-07. Standard Test Method for Potential Alkali Reactivity of Aggregates (Mortar-Bar Method), Annual Book of ASTM Standards.

[37] Köksal F., Altun F., Yiğit İ., Şahin Y. (2008). Combined Effect of Silica Fume and Steel Fiber on the Mechanical Properties of High Strength Concretes. Constr. Build. Mater..

[38] Panjehpour M., Ali A.A.A., Demirboga R. (2011). A Review for Characterization of Silica Fume and Its Effects on Concrete Properties. Int. J. Sustain. Constr. Eng. Technol..

[39] General Administration of Quality Supervision, Inspection and Quarantine, Standardization Administration Committee (2011). Sand for Construction. GB/T 14684—2011.

[40] Chen J.-L., Zhou W., Niu W., Zhang C., Li F. (2012). Explanation About Revision of Sand for Construction (Gb/T 14684-2011). Archit. Technol..

[41] Agarwal S.K. (2006). Pozzolanic Activity of Various Siliceous Materials. Cem. Concr. Res..

[42] Taguchi G. (1995). Quality Engineering (Taguchi Methods) for the Development of Electronic Circuit Technology. IEEE Trans. Reliab..

[43] Pereira-de-Oliveira L.A., Castro-Gomes J.P., Santos P.M.S. (2012). The Potential Pozzolanic Activity of Glass and Red-Clay Ceramic Waste as Cement Mortars Components. Constr. Build. Mater..

[44] Li N., Shi C., Wang Q., Zhang Z., Ou Z. (2017). Composition Design and Performance of Alkali-Activated Cements. Mater. Struct..

[45] Avet F., Scrivener K. (2018). Investigation of the Calcined Kaolinite Content on the Hydration of Limestone Calcined Clay Cement (Lc3). Cem. Concr. Res..

[46] Scrivener K., Snellings R., Lothenbach B. (2018). A Practical Guide to Microstructural Analysis of Cementitious Materials.

[47] Long S., Liu Y., Zhang F. (2010). Detection and Analysis of Cement Particle Size Distribution in Large Cement Companies in China. Cem. (Chin. J.).

[48] Alomayri T. (2017). The Microstructural and Mechanical Properties of Geopolymer Composites Containing Glass Microfibres. Ceram. Int..

[49] Afshinnia K., Rangaraju P.R. (2015). Influence of Fineness of Ground Recycled Glass on Mitigation of Alkali–Silica Reaction in Mortars. Constr. Build. Mater..

[50] Thomas M. (2011). The Effect of Supplementary Cementing Materials on Alkali-Silica Reaction: A Review. Cem. Concr. Res..

[51] Al-Zubaid A.B., Shabeeb K.M., Ali A.I. (2017). Study the Effect of Recycled Glass on the Mechanical Properties of Green Concrete. Energy Procedia.

[52] Wang Z., Shi C., Song J. (2009). Effect of Glass Powder on Chloride Ion Transport and Alkali-Aggregate Reaction Expansion of Lightweight Aggregate Concrete. J. Wuhan Univ. Technol. Mater. Sci. Ed..

[53] Urhan S. (1987). Alkali Silica and Pozzolanic Reactions in Concrete. Part 1: Interpretation of Published Results and an Hypothesis Concerning the Mechanism. Cem. Concr. Res..

[54] Islam G.M.S., Islam M.M., Akter A., Islam M.S. Green Construction Materials-Bangladesh Perspective. Proceedings of the International Conference on Mechanical Engineering and Renewable Energy 2011.

[55] Liu G., Florea M.V.A., Brouwers H.J.H. (2018). The Hydration and Microstructure Characteristics of Cement Pastes with High Volume Organic-Contaminated Waste Glass Powder. Constr. Build. Mater..

[56] Wang Y., Cao Y., Zhang P., Ma Y., Zhao T., Wang H., Zhang Z. (2019). Water Absorption and Chloride Diffusivity of Concrete under the Coupling Effect of Uniaxial Compressive Load and Freeze–Thaw Cycles. Constr. Build. Mater..

[57] Dewing E.W., Richardson F.D. (1959). Decomposition Equilibria for Calcium and Magnesium Sulphates. Trans. Faraday Soc..

[58] Scrivener K.L., Patel H.H., Pratt P.L., Parrott L.J. (1986). Analysis of Phases in Cement Paste Using Backscattered Electron Images, Methanol Adsorption and Thermogravimetric Analysis. MRS Proc..

[59] Pane I., Hansen W. (2005). Investigation of Blended Cement Hydration by Isothermal Calorimetry and Thermal Analysis. Cem. Concr. Res..

[60] Xue S., Zhang P., Bao J., He L., Hu Y., Yang S. (2019). Comparison of Mercury Intrusion Porosimetry and Multi-Scale X-Ray Ct on Characterizing the Microstructure of Heat-Treated Cement Mortar. Mater. Charact..

[61] Lu L.N., Yu S.M., He Y.J., Hu S.G. (2011). Dehydration Process of Calcium Silicate Hydrate During Heating in the Air. Adv. Mater. Res..

[62] Tajuelo Rodriguez E., Garbev K., Merz D., Black L., Richardson I.G. (2017). Thermal Stability of C-S-H Phases and Applicability of Richardson and Groves’ and Richardson C-(a)-S-H(I) Models to Synthetic C-S-H. Cem. Concr. Res..

[63] Chatterji A.K., Rawat R.S. (1965). Hydration of Portland Cement. Nature.

[64] Scrivener K.L., Nonat A. (2011). Hydration of Cementitious Materials, Present and Future. Cem. Concr. Res..

[65] Aliabdo A.A., Elmoaty A.E.M.A., Aboshama A.Y. (2016). Utilization of Waste Glass Powder in the Production of Cement and Concrete. Constr. Build. Mater..

[66] Du H., Tan K.H. (2017). Properties of High Volume Glass Powder Concrete. Cem. Concr. Compos..

[67] Shi C., Zheng K. (2007). A Review on the Use of Waste Glasses in the Production of Cement and Concrete. Resour. Conserv. Recycl..

[68] Gorospe K., Booya E., Ghaednia H., Das S. (2019). Effect of Various Glass Aggregates on the Shrinkage and Expansion of Cement Mortar. Constr. Build. Mater..

[69] Du H., Tan K.H. (2014). Concrete with Recycled Glass as Fine Aggregates. ACI Mater. J..

[70] Du H., Tan K.H. (2014). Effect of Particle Size on Alkali–Silica Reaction in Recycled Glass Mortars. Constr. Build. Mater..

